# Welfare Regimes and Out-of-Pocket Healthcare Spending in the Last Year of Life Among Cancer Decedents in Europe and Israel

**DOI:** 10.3390/cancers18182976

**Published:** 2026-09-15

**Authors:** Aviad Tur-Sinai, Netta Bentur

**Affiliations:** 1 School of Public Health, The Faculty of Social Welfare and Health Sciences, University of Haifa, Haifa 3103301, Israel; 2 The Stanley Steyer School of Health Professions, Sackler Faculty of Medicine, Tel-Aviv University, Tel Aviv-Yafo 6997801, Israel; nettabentur51@gmail.com

**Keywords:** cancer disease, out-of-pocket, economic capacity, welfare regime, SHARE

## Abstract

**Background/Objectives:** Out-of-pocket (OOP) healthcare spending can remain substantial near the end of life even in countries with broad health coverage, yet less is known about whether these expenditures follow broader welfare-regime patterns. This study examined OOP healthcare expenditure among cancer decedents and assessed whether welfare-regime differences persisted after adjustment for individual demographic and socioeconomic characteristics. **Methods:** We analyzed Survey of Health, Ageing and Retirement in Europe (SHARE) End-of-Life data for 1950 cancer decedents aged 50 years and older from 14 countries including Israel, classified into Continental, Social Democratic, Mediterranean, and East European welfare regimes. Logistic regression examined the likelihood of OOP expenditure, and linear regression examined expenditure magnitude, adjusting for gender, age, education, household economic capacity, and supplemental health insurance. **Results:** Overall, 58.0% of cancer decedents incurred OOP healthcare expenditure during their final year of life. Compared with the Continental regime, adjusted odds were higher in the Social Democratic regime (OR = 1.841) and lower in the Mediterranean (OR = 0.480) and East European (OR = 0.731) regimes. Social Democratic and East European regimes were also associated with lower expenditure levels than the Continental regime, whereas the Mediterranean regime did not differ significantly. Supplemental health insurance was associated with lower odds of OOP expenditure, while education was inversely associated with expenditure magnitude. **Conclusions:** OOP healthcare spending among cancer decedents varies systematically across welfare-regime contexts. Macro-institutional arrangements governing healthcare financing and social protection appear to be associated with household exposure to healthcare costs beyond individual socioeconomic characteristics.

## Simple Summary

Cancer care can create substantial costs for patients and families, even in countries with broad health coverage, especially during the last year of life. This study examined whether these out-of-pocket healthcare costs differ across European welfare systems and Israel, and whether such differences remain after taking patients’ demographic and socioeconomic characteristics into account. Using data on 1950 people who died from cancer in 14 countries, we found clear differences between welfare regimes in both the likelihood of paying healthcare costs directly and the amount paid. These findings suggest that financial burden near the end of life is shaped not only by individual circumstances, but also by the way healthcare and social protection systems are organized. The results may help researchers and policymakers better understand how institutional arrangements can strengthen financial protection for patients with cancer and their families.

## 1. Introduction

The economic impact of cancer treatment presents a significant challenge to healthcare systems, societies, and individuals worldwide. In recent years, European countries have witnessed a sharp increase in oncological care costs, outpacing growth in many other healthcare sectors [1,2,3]. Despite this rapid rise, oncology’s share of total health expenditure has remained relatively stable at approximately 6%, a figure that underrepresents cancer’s overall disease burden [4,5,6].

Population ageing is likely to intensify these pressures. Globally, the proportion of all cancer cases occurring among adults aged 65 years and older increased from 48.6% in 1990 to 56.4% in 2019, while their share of cancer deaths increased from 52.0% to 61.9%. Over the same period, population ageing accounted for 56.5% of the increase in cancer cases and 63.3% of the increase in cancer deaths. These trends underscore the growing importance of understanding the financial consequences of cancer care among older populations, particularly near the end of life [7].

Across Europe, healthcare systems have implemented various forms of universal coverage for essential medical services [8]. These systems differ in their approaches to government involvement, encompassing aspects such as funding mechanisms, regulatory frameworks, and service delivery models [9]. The extent of governmental contribution to healthcare spending varies significantly between nations [10]. This variation stems from a complex interplay of factors, including economic prosperity, healthcare infrastructure, pharmaceutical practices, clinical protocols, and reimbursement strategies [11]. Additionally, historical precedents, cultural norms, labor market dynamics, and political structures play crucial roles in shaping these differences [9].

Rather than treating cross-national differences in healthcare financing as isolated country characteristics, the present study draws on welfare-regime theory as a macro-institutional framework. Welfare regimes reflect broader configurations of social protection, including the relative roles of public provision, social insurance, market mechanisms, and family responsibility. Comparative European research has continued to use this framework to capture meaningful differences in the institutional and cultural environments in which economic and health-related outcomes occur [12]. Accordingly, we distinguish among four broad welfare-regime contexts: Continental, Social Democratic, Mediterranean, and East European. This approach provides an intermediate level of comparative analysis between individual characteristics and country-specific institutional arrangements and has been widely applied in comparative SHARE research, allowing countries with broadly similar social-policy orientations to be examined jointly while recognizing heterogeneity within regimes. Its purpose here is to assess whether broader configurations of social protection are systematically associated with household exposure to healthcare costs. Table 1 summarizes the broad characteristics of the four welfare regimes and the countries included in each regime in the present study.

OOP expenditure represents a particularly relevant outcome through which these institutional differences can be observed because it lies directly at the boundary between collective and private responsibility for healthcare financing. Differences in the breadth of publicly financed benefits, patient cost-sharing, pharmaceutical reimbursement, supplemental insurance, and the public financing of long-term and end-of-life care can determine whether healthcare costs are absorbed collectively or transferred to patients and their families. These institutional differences may become especially consequential during the final stages of cancer, when patients often require multiple types of healthcare simultaneously, including physician services, medications, hospitalization, and nursing or long-term care.

In the pursuit of financial sustainability and effective cost management, many healthcare systems have adopted cost-sharing strategies [18,19,20]. This approach aims to redistribute some of the financial burden from public coffers to private individuals. Consequently, out-of-pocket (OOP) expenses have become a common feature even in countries with universal healthcare or comprehensive insurance systems. Furthermore, there is a growing trend among health insurers to increase patient financial responsibility, particularly for medication costs, through higher deductibles and copayments [8]. The magnitude of OOP expenditure varies considerably across nations, ranging from 2% to 25% of the median household income in Europe [21].

The extent of OOP healthcare spending is influenced not only by national policies and regulations but also by individual patient characteristics. Factors such as age, gender, education level, marital status, economic capacity, and private insurance coverage all play significant roles in determining OOP expenses [22].

Previous research has documented substantial cross-national variation in out-of-pocket healthcare expenditure, including among patients with cancer and during the last year of life [21,23,24,25]. However, most of this literature has focused either on differences between individual countries or on patient-level socioeconomic and insurance characteristics. Less attention has been given to whether OOP expenditure follows broader macro-institutional patterns across welfare regimes, and whether these patterns persist after individual demographic and socioeconomic characteristics are taken into account. Examining OOP expenditure through a welfare-regime framework may therefore provide additional insight into how different configurations of social protection and healthcare financing shape the division of financial responsibility between public systems and households at the end of life.

Given this context, our study aimed to address three primary objectives:

1. Quantify the proportion of cancer decedents who incurred OOP healthcare expenses during their final year of life and assess the magnitude of these expenditures.
2. Examine whether the likelihood and magnitude of OOP healthcare expenditure differ systematically across welfare regimes.
3. Assess whether welfare-regime differences in OOP expenditure persist after accounting for individual demographic and socioeconomic characteristics, including supplemental health insurance.

By addressing these objectives, the study sought to clarify the extent to which household exposure to healthcare costs near the end of life reflected individual socioeconomic circumstances versus the broader institutional context in which healthcare and social protection were organized.

## 2. Methods

### 2.1. Data Source and Sample

This investigation employs a quantitative methodology, utilizing data from the Survey of Health, Aging and Retirement in Europe (SHARE-Europe), which includes Israel among its participating countries. SHARE-Europe aims to enhance our understanding of the evolving dynamics within the growing 50+ demographic and to establish a robust research foundation for evidence-based policymaking targeted at this age group.

Our analysis draws upon data from SHARE Waves 4 through 7, conducted biennially from 2011 to 2017. We supplemented this information by cross-referencing it with the SHARE End-of-Life survey, a complementary initiative undertaken at least two years after a participant’s initial involvement. This follow-up survey engaged family members of deceased SHARE participants, gathering crucial information about the circumstances surrounding the death, the decedent’s health status prior to passing, the cause of death, and healthcare expenditures incurred during the final year of life.

Through this process, we compiled a comprehensive dataset on participants who died between 2011 and 2020. Our study specifically focuses on individuals who succumbed to cancer, analyzing their experiences across four distinct welfare regime contexts.

### 2.2. Variables

#### 2.2.1. Dependent Variables

We defined two primary outcome variables based on information provided by the decedents’ family members:

1. Presence of Out-of-Pocket (OOP) Healthcare Expenditure: A binary variable indicating whether the decedent incurred any OOP expenses for healthcare services in their final year of life. These services encompassed general practitioner visits, specialist consultations, medications, hospitalizations, and nursing home care. (1 = OOP expenses incurred; 0 = no OOP expenses).
2. Total OOP Healthcare Expenditure: The cumulative amount spent out-of-pocket on all five aforementioned healthcare services during the decedent’s last twelve months, adjusted to 2020 PPP (purchasing power parity).

#### 2.2.2. Independent Variables

Our primary explanatory variable categorizes the fourteen countries in our study according to their welfare regime typology [13,14]: Continental: Austria, Germany, France, Belgium; Social Democratic: Sweden, Denmark; Mediterranean: Spain, Italy, Greece, Israel; East European: the Czech Republic, Poland, Slovenia, Estonia. This classification follows established applications of welfare-regime typologies in comparative SHARE research. In particular, previous SHARE-based studies have classified Israel together with Spain, Italy, and Greece within the Mediterranean regime and have distinguished these countries from Continental, Social Democratic, and East European welfare contexts [15].

Additional explanatory variables, derived from the initial SHARE survey in which the now-deceased individual participated, include: socio-demographic factors: gender, age, education level; socioeconomic indicators: household financial capacity (subjective self-assessment of ability to make ends meet: 1 = great difficulty; 2 = some difficulty; 3 = fairly easily; 4 = easily); possession of supplemental health insurance (1 = yes; 0 = no).

### 2.3. Ethical Considerations

This research adheres to the ethical standards set by the Max Planck Society. The SHARE project, operating under the auspices of the Max Planck Institute for Social Law and Social Policy and coordinated by the Munich Center for the Economics of Aging, has received ethical approval from the Ethics Council of the Max Planck Society.

### 2.4. Statistical Methodology

We conducted our statistical analyses using STATA Version 15.1. Our analytical approach comprised several steps:

1. Descriptive statistics: We calculated means and distributions for all variables, stratified by the presence or absence of OOP expenditure. Intergroup comparisons were performed using chi-square tests for categorical variables and *t*-tests for continuous variables.
2. Logistic regression: To identify factors associated with the likelihood of incurring OOP healthcare expenses among cancer decedents, we employed logistic regression. This analysis yielded adjusted odds ratios with corresponding 95% confidence intervals.
3. Linear regression: To explore factors influencing the magnitude of OOP healthcare expenditure, we utilized linear regression models.

For all analyses, we adopted a significance level of α = 0.05.

## 3. Results

Our investigation encompassed 1950 cancer-related deaths among individuals aged 50 and above. The deceased had a mean age of 74.4 years (SD = 9.39), with males constituting 58.0% of the sample. Educational background averaged 10.0 years of formal schooling.

Supplemental health insurance coverage was observed in approximately half (49.0%) of the cases, with a marked disparity between those who incurred out-of-pocket (OOP) expenses (53.0% had coverage) and those who did not (42.6% had coverage). Financial circumstances varied across the sample: 12.6% faced severe economic challenges, 60.8% encountered moderate financial strain, and 26.6% reported no financial difficulties.

A majority of the study population (1131 individuals, 58.0%) bore OOP healthcare costs in their final year. Those incurring such expenses exhibited higher educational attainment (mean 10.3 years) compared to their counterparts (mean 9.6 years). Paradoxically, both extreme financial hardship (13.0% vs. 12.0%) and financial stability (29.1% vs. 22.5%) were more prevalent among those with OOP expenses, suggesting a nuanced relationship between economic status and healthcare expenditure (refer to Table 2 for detailed statistics).

**Figure 1:**
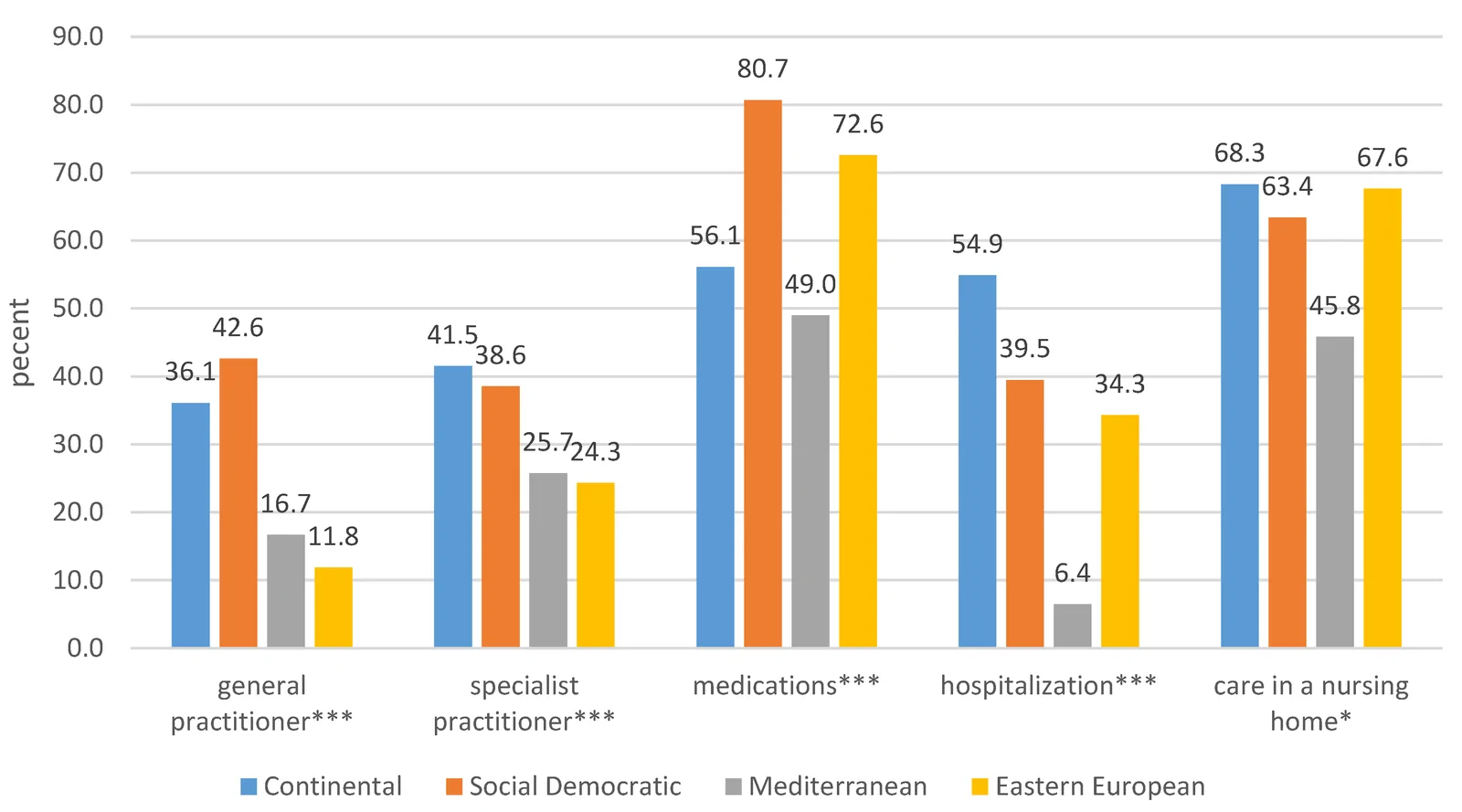
Probability of out-of-pocket expenditure on healthcare services among cancer decedents in last 12 months of life, by healthcare service and welfare regime (percent); * *p* < 0.05, *** *p* < 0.001. Note: Asterisks indicate the statistical significance of overall differences across welfare-regime groups for each healthcare service.

The analysis revealed varying probabilities of OOP expenses across different healthcare services. While general and specialist practitioner services showed moderate likelihoods, medication and nursing home care emerged as the categories most likely to incur OOP costs. Significant variations were observed across welfare regimes, with Continental and Social Democratic systems generally associated with higher OOP probabilities compared to their East European and Mediterranean counterparts. Notably, East European regimes exhibited exceptionally high probabilities for medication and nursing home care expenses (illustrated in Figure 1).

**Figure 2:**
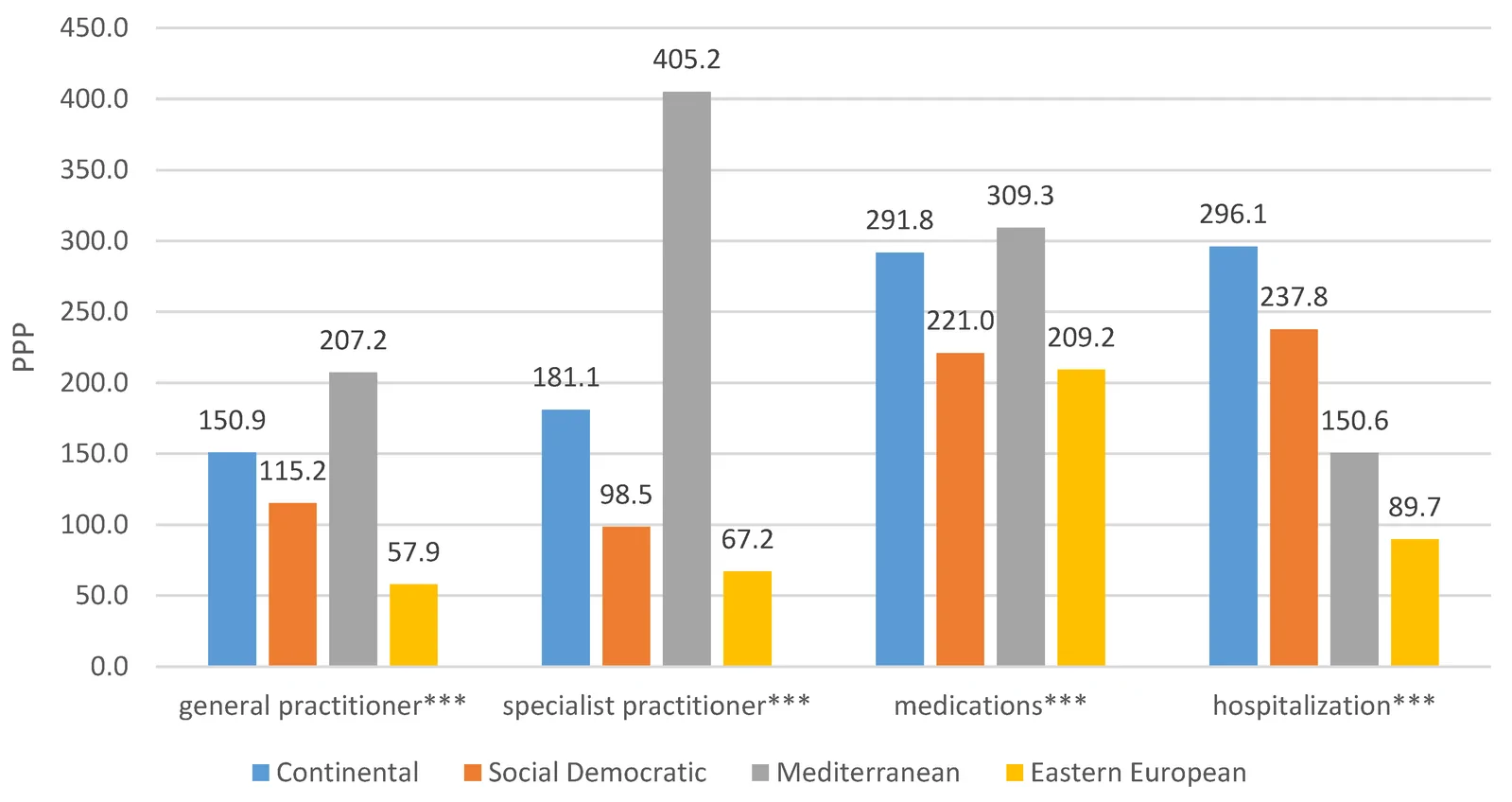
Total out-of-pocket expenditure on selected healthcare services among cancer decedents in the last 12 months of life, by healthcare service and welfare regime (PPP-adjusted values). *** *p* < 0.001; **Note:** Nursing home care is not displayed because its substantially higher expenditure levels would compress the scale and obscure differences among the other service categories. Note: Asterisks indicate the statistical significance of overall differences across welfare-regime groups for each healthcare service.

The magnitude of OOP expenses demonstrated considerable variation across both service types and welfare regimes. Nursing home care was the most expensive category, with average OOP costs ranging from 1390.7 PPP-adjusted units in East European regimes to 4900.6 in Mediterranean regimes. Because these expenditure levels were substantially higher than those observed for the other healthcare services, nursing home care was not included in Figure 2, as displaying all five categories on the same scale would substantially compress the variation among the remaining services. For general practitioner visits, specialist care, medications, and hospitalization, East European regimes consistently displayed the lowest average OOP expenses. Mediterranean regimes had the highest average OOP costs for most of these services, with the exception of hospitalization, for which Continental regimes showed the highest expenditure (Figure 2).

Logistic regression analyses (Table 3) examined factors associated with the likelihood of incurring OOP expenses. Model 1, which included individual characteristics only, showed that higher education (OR = 1.036, 95% CI = 1.012–1.060, *p* < 0.01) and greater economic capacity (OR = 1.112, 95% CI = 1.006–1.229, *p* < 0.05) were associated with higher odds of OOP spending, whereas supplemental health insurance was associated with lower odds (OR = 0.901, 95% CI = 0.858–0.946, *p* < 0.001). Model 2, which included welfare regime only, showed significantly higher odds of OOP expenditure in the Social Democratic regime (OR = 1.742, 95% CI = 1.198–2.532, *p* < 0.01) and significantly lower odds in the Mediterranean regime (OR = 0.447, 95% CI = 0.344–0.581, *p* < 0.001) compared with the Continental regime. The East European regime did not differ significantly from the Continental regime in Model 2 (OR = 0.887, 95% CI = 0.686–1.146). In Model 3, which incorporated both individual characteristics and welfare regime, supplemental health insurance remained associated with lower odds of OOP expenditure (OR = 0.894, 95% CI = 0.846–0.946, *p* < 0.001). Compared with the Continental regime, the odds of OOP expenditure were significantly higher in the Social Democratic regime (OR = 1.841, 95% CI = 1.251–2.709, *p* < 0.01) and significantly lower in both the Mediterranean (OR = 0.480, 95% CI = 0.362–0.635, *p* < 0.001) and East European regimes (OR = 0.731, 95% CI = 0.548–0.975, *p* < 0.05).

Linear regression analyses of OOP expenditure amounts (Table 4) yielded additional insights. Model 1 indicated an inverse relationship between educational attainment and OOP expenses. Model 2 revealed no significant disparity between Continental and Mediterranean regimes but highlighted substantial differences with other systems. Model 3, which integrated patient characteristics and welfare regimes, corroborated these findings: Social Democratic and East European regimes demonstrated significantly lower OOP expenses compared to Continental systems. Education persisted as a significant predictor, maintaining its negative association with OOP expense magnitudes.

## 4. Discussion

The principal finding of this study is not simply that OOP expenditure differs across countries, but that both the likelihood and magnitude of expenditure display systematic variation across broader welfare-regime contexts. Importantly, these associations remained after adjustment for individual demographic and socioeconomic characteristics. This finding supports the relevance of viewing household exposure to healthcare costs within the macro-institutional context in which healthcare and social protection are organized, rather than interpreting OOP expenditure solely as the product of individual economic resources or insurance status.

Welfare-regime differences should therefore not be interpreted as the effect of a single institutional characteristic. Rather, regime membership captures constellations of policies concerning public financing, insurance, eligibility, cost-sharing, pharmaceutical reimbursement, long-term care, and the relative responsibilities assigned to households and the state. Such policies tend to coexist and may jointly influence whether healthcare needs generate private expenditure. The regime framework is therefore useful precisely because it captures these broader institutional configurations while allowing individual-level characteristics to be considered simultaneously [12].

The pattern across welfare regimes was not identical for the occurrence and magnitude of OOP expenditure. Relative to the Continental regime, the Social Democratic regime was associated with higher odds of incurring any OOP expenditure but with lower expenditure levels, whereas the Mediterranean regime was associated with lower odds of OOP expenditure but did not differ significantly in expenditure magnitude. The East European regime was associated with both lower adjusted odds and lower expenditure levels. This divergence suggests that institutional arrangements may shape exposure to any private payment differently from the amount ultimately paid. Some systems may expose a larger proportion of patients to relatively limited cost-sharing while providing greater protection against high expenditures, whereas others may concentrate larger payments among a smaller share of patients. Because the present data do not identify the specific financing mechanisms underlying these patterns, these interpretations should be regarded as hypotheses for future research.

As anticipated, OOP expenditure was least likely for general practitioner and specialist services and most likely for medications and nursing care. This pattern is not surprising since most European countries, even those with comprehensive health coverage, often require cost-sharing for medications. Additionally, long-term inpatient care is typically only covered for the very poor in many countries [26]. Differences in OOP expenditure on medications may stem from variations in how countries manage the reimbursement processes for medications [27]. Even when a new drug is deemed promising, different countries implement diverse policies regarding its pricing and reimbursement, leading to substantial disparities in medication access [28,29].

Differences in OOP expenditure on inpatient nursing care may be explained by varying eligibility criteria for such care across different countries. While most countries with Social Democratic and Continental regimes offer various forms of public and private long-term care insurance [30,31], these insurance options are almost nonexistent in countries following the East European method [26].

When we analyzed the probability of OOP expenditure based on patient characteristics, the only factor significantly associated with the likelihood of incurring OOP expenses, after factoring in welfare regimes, was having supplemental health insurance. Gender, age, education, and economic capacity did not show significant associations with OOP expenditure. This contrasts with other studies that found a relationship between OOP spending and socio-demographic characteristics, particularly the presence of supplemental insurance [28,32]. Specifically, the presence of supplemental insurance was found to significantly influence the extent of OOP spending, corroborating findings from previous research [33,34]. The attenuation of several individual-level associations after welfare regime was introduced suggests that the relationship between personal resources and OOP expenditure is embedded within, and potentially conditioned by, the broader institutional environment.

This study has several limitations. First, the data are based on self-assessed OOP expenditure on healthcare, which may lead to under- or over-estimation. Second, the findings are based on proxies for deceased patients, potentially resulting in memory problems and recall or social-desirability biases. However, interviewing bereaved families to explore care up to the end of life is common in research [35], and studies have shown that using proxy respondents is particularly appropriate when recall bias is mitigated by some objective measure of expenditure, such as receipts [36]. Third, our respondents were sampled from households, meaning individuals without relatives, particularly those who died in nursing institutions, were not included. Fourth, the small sample size under each welfare regime might have affected the proportions. Fifth, other health conditions and comorbidities that could influence OOP expenditure were not included in the study. In addition, OOP expenditure during the final 12 months of life may partly reflect care pathways and institutional conditions established earlier in the course of the disease. These may include the availability and implementation of clinical guidelines for cancer management, access to palliative and hospice care, and broader aspects of oncology and healthcare infrastructure. Such information is not captured in SHARE and therefore could not be incorporated into the present analysis. Future research incorporating more detailed indicators of cancer-care organization and treatment pathways may help identify the specific mechanisms underlying the observed differences in OOP expenditure. Finally, welfare-regime classifications necessarily simplify complex national institutional arrangements. Countries within the same regime may differ in healthcare financing, benefit design, economic resources, reimbursement policies, and cultural expectations. The regime coefficients should therefore be interpreted as associations with broad macro-institutional configurations rather than as evidence that all countries within a regime are homogeneous or that regime membership itself is causal.

## 5. Conclusions

The findings suggest that the financial consequences of healthcare use near the end of life are shaped not only by patients’ individual socioeconomic resources but also by the broader institutional context in which healthcare and social protection are organized. Welfare regimes provide a useful comparative framework for identifying systematic differences in household exposure to out-of-pocket healthcare expenditure across countries with different configurations of social protection.

Rather than implying that one welfare model is uniformly superior to another, the results highlight the importance of examining how specific components of health and social policy—including cost-sharing arrangements, pharmaceutical reimbursement, supplemental insurance, and the financing of long-term and end-of-life care—distribute financial responsibility between public systems and households. Greater attention to these institutional mechanisms may help policymakers identify where financial protection can be strengthened for patients with cancer and their families during the final stage of life.

## Figures and Tables

**Table 1:** Broad characteristics of welfare regimes and countries included in the present study.

Welfare Regime	Broad Characteristics	Countries Included in the Present Study
Social Democratic	Universalistic and comparatively generous welfare provision, characterized by broad eligibility, extensive public benefits, and a strong role of the state in social protection.	Sweden, Denmark
Continental	Substantial public and social-insurance provision, representing an intermediate position between the more universalistic Social Democratic model and the more family-oriented Mediterranean model.	Austria, Germany, France, Belgium
Mediterranean	More restrictive public provision and a stronger familistic orientation, with greater reliance on family responsibility and informal support.	Spain, Italy, Greece, Israel
East European	Post-socialist and comparatively heterogeneous welfare arrangements, combining elements of public provision with more constrained resources and stronger reliance on family responsibility.	Czech Republic, Poland, Slovenia, Estonia

**Note:** The characteristics presented are broad ideal-typical features of welfare regimes and do not imply institutional homogeneity among countries within each regime. Country allocation refers to the countries included in the present analytical sample. **Source:** Adapted from Esping-Andersen [13,14] and previous comparative SHARE-based applications of welfare-regime typologies [15,16,17].

**Table 2:** Characteristics of cancer decedents (percent).

		No OOP Spending (*n* = 819, 42.00%)	OOP Spending (*n* = 1131, 58.00%)	F/χ^2^	ALL (*n* = 1950)
Gender	Female	43.11	41.31	0.61	42.00
	Male	56.89	58.69		58.00
Age (mean and SD)		74.19 (9.63)	74.54 (9.23)	0.62	74.40 (9.39)
Education (mean and SD)		9.61 (4.59)	10.28 (4.24)	10.75 **	10.02 (4.39)
Economic capacity (household’s ability to make ends meet)	With great difficulty	12.01	12.98	11.95 **	12.60
	With some difficulty	31.92	28.16		29.60
	Fairly easily	33.56	29.77		31.22
	Easily	22.51	29.07		26.57
Supplemental health insurance	No supplemental insurance	57.39	47.03	19.70 ***	51.01
	Has supplemental health-insurance coverage	42.61	52.97		48.99

** *p* < 0.01, *** *p* < 0.001. Note: Values are percentages unless otherwise indicated. OOP—out-of-pocket; SD—standard deviation; χ^2^—chi-square statistic.

**Table 3:** Probability of out-of-pocket expenditure on healthcare services among cancer decedents (odds ratios) (95% CI) (Logit model).

	Model 1		Model 2		Model 3	
	OR (SE)	95% CI	OR (SE)	95% CI	OR (SE)	95% CI
Male	1.019 (0.10)	(0.840, 1.237)			1.080 (0.11)	(0.887, 1.315)
	
Age	1.004 (0.01)	(0.993, 1.015)			1.004 (0.01)	(0.993, 1.015)
	
Education	1.036 ** (0.01)	(1.012, 1.060)			1.015 (0.01)	(0.991, 1.040)
	
Economic capacity (1 = makes ends meet with great difficulty, 4 = easily)	1.112 * (0.06)	(1.006, 1.229)			0.981 (0.05)	(0.880, 1.093)
	
supplemental Health insurance (1 = yes, 0 = no)	0.901 *** (0.02)	(0.858, 0.946)			0.894 *** (0.03)	(0.846, 0.946)
Continental			Ref.		Ref.	
Social Democratic			1.742 ** (0.33)	(1.198, 2.532)	1.841 ** (0.360)	(1.251, 2.709)
Mediterranean			0.447 *** (0.06)	(0.344, 0.581)	0.480 *** (0.07)	(0.362, 0.635)
East European			0.887 (0.12)	(0.686, 1.146)	0.731 * (0.11)	(0.548, 0.975)
Constant	1.011 (0.45)	(0.425, 2.407)	2.381 *** (0.25)	(1.946, 2.913)	2.227 (1.06)	(0.873, 5.680)
*n*	1901		1950		1901	
Log-likelihood	−1209.1198		−1217.0832		−1180.2169	

* *p* < 0.05, ** *p* < 0.01, *** *p* < 0.001. Note: OR—odds ratio; CI—confidence interval; Ref.—reference category; SE—standard error.

**Table 4:** Out-of-pocket expenditure on healthcare services among cancer decedents, OLS regression model (dependent variable: ln(out-of-pocket expenditure)).

	Model 1		Model 2		Model 3	
	Coef. (SE)	95% CI	Coef. (SE)	95% CI	Coef. (SE)	95% CI
Male	−0.022 (0.08)	(−0.182, 0.137)			−0.039 (0.08)	(−0.198, 0.120)
	
Age	−0.008 (0.00)	(−0.016, 0.001)			−0.008 (0.00)	(−0.017, 0.001)
	
Education	−0.031 ** (0.01)	(−0.050, −0.012)			−0.022 * (0.01)	(−0.041, −0.002)
	
Economic capacity (1 = makes ends meet with great difficulty, 4 = easily)	−0.045 (0.04)	(−0.126, 0.036)			−0.031 (0.04)	(−0.118, 0.057)
	
supplemental Health insurance (1 = yes, 0 = no)	0.062 ** (0.02)	(0.021, 0.102)			0.033 (0.02)	(−0.011, 0.078)
	
Continental			Ref.		Ref.	
Social Democratic			−0.286 * (0.13)	(−0.538, −0.034)	−0.261 * (0.13)	(−0.522, −0.000)
	
Mediterranean			0.204 (0.12)	(−0.024, 0.432)	0.169 (0.12)	(−0.071, 0.408)
	
East European			−0.354 *** (0.10)	(−0.556, −0.152)	−0.292 ** (0.11)	(−0.513, −0.072)
	
Constant	5.574 *** (0.37)	(4.847, 6.302)	4.872 *** (0.08)	(4.716, 5.029)	5.651 *** (0.39)	(4.886, 6.416)
*n*	1241		1277		1241	
Adj. R^2^	0.0164		0.0225		0.0295	

* *p* < 0.05, ** *p* < 0.01, *** *p* < 0.001. Note: OLS—ordinary least squares; CI—confidence interval; Ref.—reference category; SE—standard error.

## Data Availability

The datasets analyzed during the current study are available in the Survey of Health Ageing and Retirement in Europe (SHARE) repository, https://share-eric.eu/, accessed on 12 August 2026.
